# Histone lactylation-mediated glycolysis-ferroptosis axis in neurological diseases

**DOI:** 10.3389/fnmol.2026.1820758

**Published:** 2026-07-03

**Authors:** Zengrong Wei, Lu Bai, Xueting Liu, Yanling Zhou, Lei Zheng, Baiwen Zhang, Long Wang, Wei Zou

**Affiliations:** 1 The First Clinical Medical College, Heilongjiang University of Chinese Medicine, Harbin, China; 2 Shenzhen Bao’an Authentic TCM Therapy Hospital, Shenzhen, China; 3 First Affiliated Hospital, Heilongjiang University of Chinese Medicine, Harbin, China

**Keywords:** epigenetics, ferroptosis, glycolysis, histone lactylation, neurological diseases

## Abstract

Histone lactylation is an emerging epigenetic modification that covalently links the glycolytic metabolite lactate to histones, thereby establishing a direct link between cellular metabolic status and gene transcription programs. Recent studies have shown that this modification plays a key role in regulating cellular sensitivity to ferroptosis, forming a novel regulatory axis of “glycolysis-lactylation-ferroptosis.” This article systematically reviews the biological functions of histone lactylation in the nervous system, with a focus on elucidating how it participates in the pathological processes of various neurological diseases, including Alzheimer’s disease (AD), Parkinson’s disease (PD), cerebral stroke, and amyotrophic lateral sclerosis (ALS), by regulating the expression of ferroptosis-related genes. The article integrates the latest research on molecular mechanisms, explores the value of this regulatory axis as a potential biomarker for disease diagnosis and a therapeutic target, and provides an outlook on future research directions in this field.

## 1 Introduction

Neurological diseases, including neurodegenerative diseases and acute brain injury, have complex pathological mechanisms involving various processes such as metabolic disorders, oxidative stress, inflammation, and cell death. Among these processes, neuroinflammation and oxidative stress are core factors driving neuronal damage and dysfunction. In AD, the deposition of β-amyloid protein (Aβ) is considered an important pathological marker (Meng et al., 2022). Its upstream initiation mechanism is often directly related to the abnormal accumulation of transition metals such as unstable iron, which can participate in disease initiation and progression by promoting oxidative stress responses and protein misfolding. In this context, the accumulation of Aβ may reflect a response to local metal ion imbalances. Under conditions of metabolic reprogramming, lactate accumulation can lead to a decrease in microenvironmental pH, inducing the protonation of histidine residues, thereby promoting the release of metal ions from protein binding sites. At the same time, Aβ peptides can act as metal ion buffering molecules in the synaptic microenvironment, regulating transient increases in free metal ions to some extent. However, this process may also promote their own aggregation and exacerbate toxic effects. In PD, similar disruptions in metal ion homeostasis are also considered one of the key pathological foundations. Abnormal iron deposition can enhance oxidative stress through the Fenton reaction and interact with mitochondrial dysfunction and persistent neuroinflammation, collectively promoting damage to dopaminergic neurons (Kartik et al., 2023). Additionally, α-synuclein also has the ability to bind metal ions under physiological conditions and may be involved in the regulation of local metal homeostasis at synapses. However, under pathological conditions, its abnormal aggregation may similarly originate from or exacerbate metal ion imbalances. These pathological processes are not linear causal relationships but are more likely to manifest as a complex interaction network between metabolic abnormalities, disruption of metal ion homeostasis, protein aggregation, and oxidative stress. They mutually amplify through positive feedback mechanisms, thereby accelerating the progression of neurodegenerative diseases (Asveda et al., 2023).

In recent years, ferroptosis has been confirmed to be widely involved in neuronal damage in various neurological diseases. The characteristics of ferroptosis include intracellular iron overload, glutathione (GSH) depletion, and toxic accumulation of lipid peroxides (Lan et al., 2023). In various central nervous system (CNS) disease models, such as spinal cord injury (SCI), cerebral stroke, and neurodegenerative diseases, neuronal ferroptosis has been observed (Huang et al., 2025). After SCI, ferroptosis not only directly leads to neuronal death but also exacerbates secondary neuroinflammation, hindering neurological recovery (Wang et al., 2025a). Inhibiting ferroptosis has been shown to effectively reduce neuronal damage and improve functional outcomes, making it a highly promising therapeutic target (Ge et al., 2022). Meanwhile, reprogramming of energy metabolism in the brain, particularly the enhancement of glycolysis, is a hallmark feature of glial cell activation in neuroinflammation and disease states, producing large amounts of lactate (Vizuete and Gonçalves, 2024). In neuroinflammatory environments, microglia and astrocytes are activated, shifting their metabolic mode from oxidative phosphorylation (OXPHOS) to glycolysis to rapidly generate ATP and synthesize biomacromolecules, supporting their pro-inflammatory phenotype and function (Qin et al., 2025a). The lactate produced by this metabolic shift is not only a product of energy metabolism but has also been found to act as a signaling molecule, directly regulating gene expression through histone lactylation modification (Chen K. et al., 2026). Studies have shown that in autism spectrum disorder models, microglial activation is accompanied by upregulation of key glycolytic enzyme expression and increased lactate levels, while intervention in glycolysis can improve behavioral phenotypes (Qin et al., 2025a). This suggests that increased glycolytic flux and its product, lactate, may profoundly influence cell death fate, including ferroptosis, through epigenetic mechanisms.

Existing evidence indicates a close interplay between metabolism and cell death pathways. Lactate produced by glycolysis may regulate cellular sensitivity to ferroptosis by affecting the cellular redox state or directly modifying key proteins involved in ferroptosis regulation (Chen K. et al., 2026). The lipid peroxidation and membrane integrity disruption caused by ferroptosis itself may also inversely affect cellular energy metabolism homeostasis (Huang et al., 2025). Therefore, elucidating the mechanism of histone lysine lactylation in this interaction network is crucial for understanding the pathogenesis of neurological diseases. This review will delve into how histone lysine lactylation integrates glycolytic metabolic flux with ferroptosis signaling pathways, providing a detailed analysis of its mechanisms, current research status in specific neurological diseases, and the clinical translation prospects of novel therapeutic targets. By outlining the glycolysis-histone lysine lactylation-ferroptosis axis, it offers a theoretical basis for therapies targeting the intersection of metabolism, epigenetics, and cell death.

## 2 Histone lactylation: the core mechanism linking metabolism and epigenetics

### 2.1 The chemical nature of histone lactylation and the “writer-eraser” enzyme system

Histone lactylation is a recently discovered post-translational modification of proteins, specifically referring to the covalent attachment of lactate to the ε-amino group of histone lysine residues, forming ε-N-lactyllysine (Peng and Du, 2025). The chemical essence of this modification lies in the fact that lactate molecules are activated to lactyl-CoA by specific enzymes, and subsequently, the lactyl group is transferred to specific lysine sites on histones by “writer” enzyme. The N-terminal tails of histones (especially H3 and H4) are rich in lysine residues, and these tail structures are disordered, protruding from the nucleosome, exhibiting high pliability and accessibility (Luger et al., 1997). This spatial exposure characteristic makes the N-terminal lysines more easily recognized and modified by lactyltransferases, thereby becoming the main region for lactylation (Sabari et al., 2017). In fact, currently identified histone lactylation sites (such as H3K9, H3K18, H3K56, etc.) are all located in exposed regions near the N-terminal tails or core areas, validating this structure-function association. More importantly, the disordered N-terminal chains of H3/H4 histones are not only “hotspots” for modifications but also core hubs for chromatin regulation of gene expression. These loosely disordered segments can interact with various transcription factors, chromatin remodeling complexes, and modification “reader” proteins, dynamically regulating the compaction state of chromatin and gene accessibility (Zentner and Henikoff, 2013). When lactate accumulation leads to elevated histone lactylation levels, the charge and conformation of these N-terminal chains change, thereby affecting their binding with DNA or other regulatory proteins, ultimately reshaping the gene expression profile. Histone acetyltransferases, among others, can utilize lactyl-CoA as a substrate to covalently attach the lactyl group to sites such as H3K9, H3K18, and H3K56 (Rejili, 2025; Zhang Y. et al., 2026). Lactate exists in two stereoisomers, L- and D-, derived from different metabolic precursors. L-lactate primarily originates from glycolysis, while D-lactate may be generated through non-enzymatic reactions in the glyoxalase pathway (Moreno-Yruela et al., 2022a). Techniques like high-resolution ion mobility spectrometry can effectively distinguish these isomers, revealing the fine structure of histone modifications (Berthias et al., 2026). The “erasure” of histone lactylation is carried out by delactylases. Research indicates that members of the histone deacetylase (HDAC) family, particularly HDAC1-3, are potent histone delactylases capable of specifically removing lactyl groups from histones (Dai et al., 2022). Additionally, NAD+-dependent sirtuin family deacylases, such as SIRT1, SIRT2, and SIRT3, have also been confirmed to possess varying degrees of delactylation activity (Fan et al., 2023). SIRT3 has been identified as a specific “eraser” for H4K16-la and H3K9-la, and its crystal structure reveals its binding mechanism with lactylated peptides (Chen et al., 2025). This dynamic regulatory system allows histone lactylation levels to be precisely adjusted according to cellular state. The level of this modification is directly influenced by intracellular lactate concentration, thereby translating changes in the metabolic state of the cellular microenvironment into epigenetic signals at the chromatin level (Sharma and Pal, 2021). Under pathological conditions such as the tumor microenvironment, enhanced glycolysis leads to significant lactate accumulation, increased histone lactylation levels, remodeling of gene expression profiles, and affecting various biological processes like cell proliferation and immune responses (Zhang K. et al., 2026). Therefore, histone lactylation acts as a metabolic sensor, playing a key role in linking cellular metabolism and epigenetic regulation. It is noteworthy that this concept was first proposed by Zhang et al. (2019), who discovered that histone lysine lactylation in macrophages can directly activate genes involved in wound healing, opening up a new field of metabolic modification regulating gene expression. Subsequently, Moreno-Yruela et al. (2022b) further identified histone delactylases, completing the dynamic regulatory mechanism of this modification.

Lactylation can specifically regulate target gene expression rather than inducing widespread chromatin relaxation, primarily due to the following mechanisms: First, this modification is catalyzed by specific lactyltransferases (e.g., p300/CBP), whose substrate recognition depends on the local chromatin environment and protein interactions, ensuring that the modification occurs in the promoter or enhancer regions of specific genes rather than being randomly distributed. Second, lactylation often synergizes or antagonizes with other epigenetic modifications such as acetylation and methylation, collectively forming a “modification code” that limits its scope of influence by recruiting specific transcription factors or counteracting inhibitory modifications. Furthermore, chromatin itself is highly structurally differentiated, and lactylation mostly occurs in already partially open euchromatin regions, while the tight packaging of heterochromatin and the constraints of local auxiliary factors further prevent the spread of the modification. Meanwhile, as a cellular metabolite, lactate concentration increases locally under conditions such as hypoxia or inflammation, causing the modification to be activated only in metabolically active specific signaling pathways, achieving “on-demand regulation.” Finally, lactylation also modifies non-histone proteins such as transcription factors and metabolic enzymes, indirectly and precisely regulating gene expression networks by altering the functions of these proteins. These mechanisms collectively ensure that lactylation acts as a “precise key,” achieving precise regulation of specific genes while maintaining the overall structural stability of chromatin.

### 2.2 Regulation of histone lactylation by glycolytic flux

In neurological diseases, activated microglia and astrocytes exhibit significant metabolic reprogramming, characterized by enhanced glycolytic flux. This metabolic shift aims to rapidly generate ATP and synthesize biomacromolecules to cope with inflammatory or injury stress, but it also leads to substantial lactate accumulation (Qin et al., 2025b). After SCI, the expression of the key glycolytic regulator PFKFB3 in astrocytes is significantly upregulated to support neuronal energy supply, and the absence of PFKFB3 exacerbates neuronal ferroptosis and hinders functional recovery (Xiong et al., 2025). This enhancement of glycolysis is not limited to glial cells; under specific stress conditions, neurons may also undergo metabolic reprogramming, and the dynamic changes in their lactylation modifications are closely related to cell survival or death decisions (Wang Y. et al., 2024). Studies show that after cerebral ischemia-reperfusion injury (CIRI), a high-glycolysis subpopulation appears in microvascular endothelial cells, characterized by mitochondrial dysfunction and necroptosis activation, a process associated with lactate accumulation and increased histone lactylation (Zhou et al., 2026). The accumulated lactate is not merely an end product of glycolysis but serves as a key precursor that directly elevates histone lactylation levels, thus forming a positive feedback axis linking metabolism and epigenetics. In osteoarthritis (OA), IL-1β-stimulated chondrocytes also exhibit increased glycolytic flux, lactate production, and elevated levels of histone H4K12 lactylation. Lactate-induced H4K12 lactylation promotes pro-inflammatory gene transcription and reinforces the PFKFB3-mediated positive feedback loop maintaining glycolysis and inflammation (He et al., 2025). In AD, H4K12-la levels are elevated in microglia adjacent to Aβ plaques. This lactate-dependent histone modification enriches at the promoter regions of glycolytic genes and activates their transcription, forming a glycolysis/H4K12-la/PKM2 positive feedback loop that exacerbates microglial dysfunction in the disease (Pan et al., 2022). This feedback mechanism is prevalent in various pathological contexts. For example, in diabetic nephropathy, lactate-promoted histone H4K12 lactylation enhances RUNX1 transcription, and RUNX1 subsequently activates HK1 and SLC2A1, thereby accelerating glycolysis and renal fibrosis (Shen et al., 2026).

Under specific stress conditions such as ischemia, hypoxia, or neurodegeneration, neurons may undergo metabolic reprogramming. The dynamic changes in lactylation modifications are tightly linked to neuronal survival/death decisions. In CIRI, lactate overload increases histone lactylation, and reduced expression of SMEK1 in microglia is associated with increased lactate levels and exacerbated neuroinflammation (Si et al., 2024). Mechanistically, microglia-specific knockout of SMEK1 enhances lactate production by inhibiting the PDK3-PDH pathway, increases H3K9-la, thereby activating transcription of lactate dehydrogenase A (LDHA) and hypoxia-inducible factor-1α (Hif-1α) and promoting glycolysis (Si et al., 2024). Furthermore, during the early reperfusion phase of myocardial ischemia/reperfusion injury, aerobic glycolytic flux in cardiomyocytes is crucial for maintaining histone H3 lactylation and cell survival. HSPA12A maintains appropriate glycolytic activity by stabilizing Hif1α, thereby supporting H3 lactylation and improving cardiomyocyte survival (Yu et al., 2024). These findings indicate that lactate production driven by glycolytic flux and subsequent histone lactylation are core epigenetic mechanisms by which glial cells and neurons coordinate inflammatory responses, energy metabolism, and cell fate decisions under disease conditions. Targeting this metabolic-epigenetic axis, for example by inhibiting key glycolytic enzymes or histone lactylation modifications, may provide new strategies for treating neurological diseases (Zhang Y. M. et al., 2025).

## 3 The key role of ferroptosis in neurological diseases

### 3.1 The core biochemical pathway and regulatory factors of ferroptosis

**FIGURE 1:**
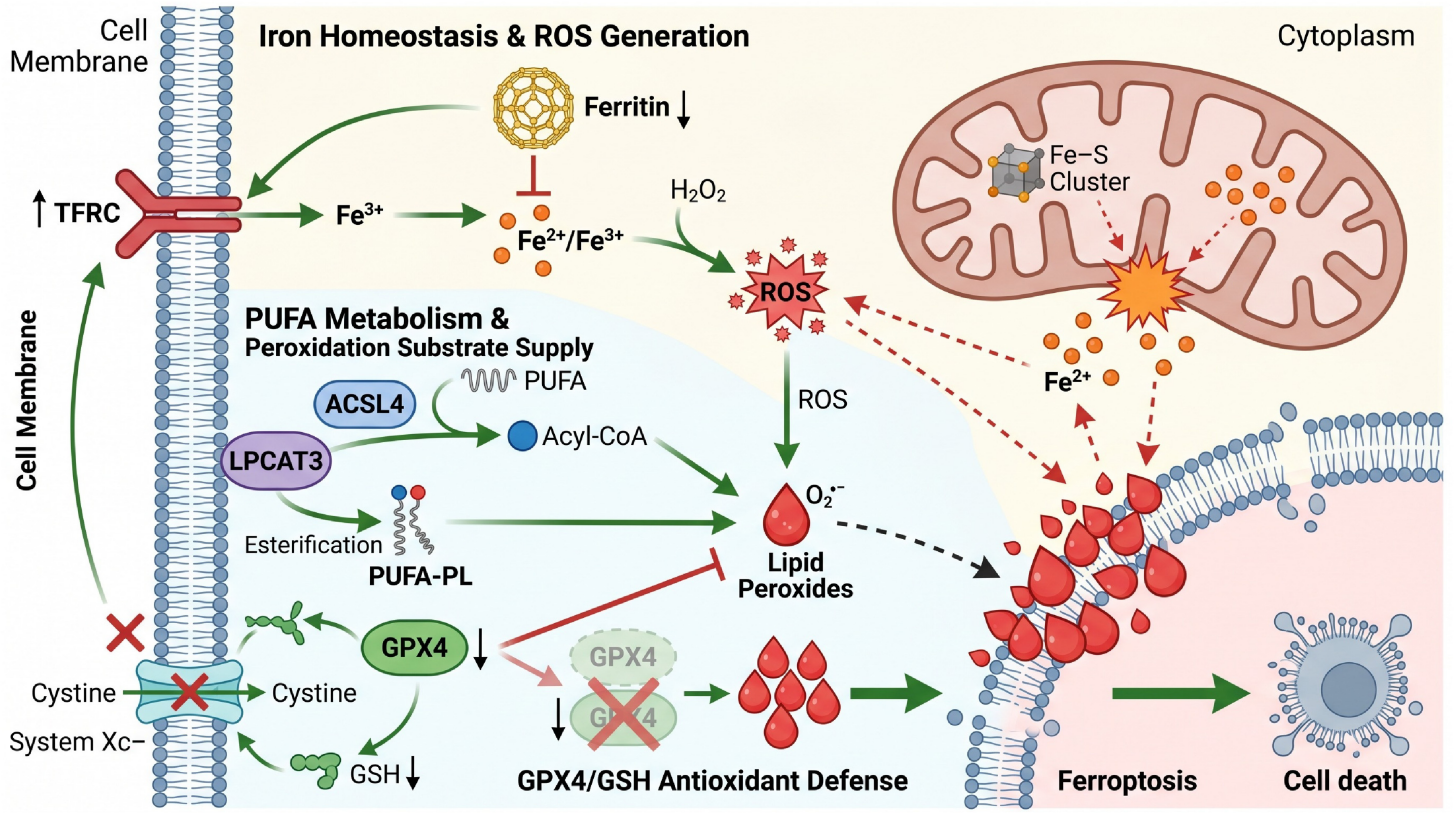
This schematic illustrates the core regulatory mechanisms of ferroptosis. (1) Dysregulated iron homeostasis: TFRC mediates Fe^3 +^ uptake, while reduced ferritin releases Fe^2 +^; damaged mitochondrial Fe-S clusters further increase labile Fe^2 +^, which drives ROS generation via the Fenton reaction. (2) Lipid peroxidation substrate supply: ACSL4 and LPCAT3 promote PUFA activation and esterification into membrane phospholipids (PUFA-PL), providing substrates for peroxidation. (3) Impaired antioxidant defense: dysfunction of System Xc^–^ leads to GSH depletion and reduced GPX4 activity, diminishing the capacity to detoxify lipid peroxides. These processes converge to cause excessive lipid peroxide accumulation, membrane rupture, and ultimately ferroptotic cell death.

Ferroptosis is a form of regulated cell death mediated by unstable iron ions. Its essence is the toxic accumulation of iron-dependent lipid peroxides, particularly phospholipid hydroperoxides, which triggers cell death when this accumulation exceeds the clearance capacity of the cellular antioxidant defense system (Tang and Kroemer, 2020). Unstable iron ions (such as iron loosely bound to low-molecular-weight ligands) are the core condition for catalyzing the Fenton reaction, and their high reactivity can efficiently convert hydrogen peroxide (H_2_O_2_) into highly toxic hydroxyl radicals (∙OH), generating a large amount of reactive oxygen species (ROS) that initiate the peroxidation of polyunsaturated fatty acids (PUFAs), ultimately leading to the disruption of cell membrane structure (Hadian and Stockwell, 2020). Glutathione peroxidase 4 (GPX4) is the key executor in the defense system. It utilizes reduced GSH as a substrate to specifically reduce phospholipid hydroperoxides, thereby maintaining the integrity of the cell membrane (Song and Long, 2020). When the activity of GPX4 is inhibited or its expression is downregulated, the clearance capacity for lipid peroxides is severely impaired, and the cell proceeds toward ferroptosis (Wang et al., 2020). This pathway exhibits significant differences from classical forms of cell death such as apoptosis and necrosis in terms of morphology, biochemistry, and genetics, constituting its unique regulatory network (Zhang Y. et al., 2022; Figure 1).

The key negative regulators of ferroptosis form a synergistic defense network. In addition to GPX4, which directly reduces lipid peroxides, the cystine/glutamate antiporter is a crucial upstream link for maintaining intracellular antioxidant capacity by taking up cystine to synthesize GSH, providing the essential reducing power for GPX4 (Yang et al., 2025). Iron metabolism-related proteins finely regulate the level of the labile iron pool within cells to prevent its excessive accumulation and the catalysis of harmful lipid peroxidation. For example, ferritins reduce the concentration of the labile iron pool by storing iron, thereby inhibiting the occurrence of ferroptosis (Xie Z. et al., 2025. Conversely, transferrin receptor 1 (TFRC) is responsible for iron uptake into cells, and its upregulation may increase cellular sensitivity to ferroptosis (Cao Y. et al., 2025). Studies have shown that in atherosclerosis models, ferroptosis is closely related to the dysregulation of the TFR1/SLC11A2/GPX4 signaling pathway, exacerbating lipid metabolism disorders (Cao Y. et al., 2025). Furthermore, nuclear factor erythroid 2-related factor 2 (Nrf2), as an important transcription factor, plays a central role in combating ferroptosis by upregulating the expression of various antioxidant genes, including GPX4 (Nishizawa et al., 2023). The key positive regulators of ferroptosis drive the amplification of lipid peroxidation signals. PUFAs, particularly arachidonic acid and adrenic acid, are the primary substrates for lipid peroxidation (Liu J. et al., 2022). Long-chain acyl-CoA synthetase 4 (ACSL4) and lysophosphatidylcholine acyltransferase 3 (LPCAT3) are key enzymes in the synthesis and esterification of PUFAs. ACSL4 is responsible for activating free PUFAs into acyl-CoAs, while LPCAT3 esterifies them into membrane phospholipids, providing abundant substrates for subsequent peroxidation reactions (Bi et al., 2024). Studies indicate that in a pressure overload-induced heart failure model, the expression of ACSL4 in cardiomyocytes is significantly upregulated, and its overexpression exacerbates cardiac dysfunction by promoting ferroptosis (Bi et al., 2024). Lipid peroxidases, such as members of the arachidonate lipoxygenase family, can directly catalyze the peroxidation of PUFAs in membrane phospholipids and are the direct effector molecules executing lipid peroxidation damage (Maheshwari, 2023). The activity of these positive regulators collectively determines the sensitivity of the cell membrane to peroxidative damage, constituting the core biochemical engine for the execution of ferroptosis.

### 3.2 Evidence of ferroptosis involvement in the pathology of neurological diseases

Ferroptosis plays a key role in the pathological processes of various neurological diseases (Li et al., 2024b). Its core mechanism involves iron homeostasis imbalance, GSH depletion, and decreased GPX4 activity, ultimately leading to the accumulation of lethal lipid peroxides (Yao et al., 2021). In disease models such as neurodegenerative diseases and ischemic stroke, specific markers of ferroptosis, including elevated lipid peroxidation levels, reduced GPX4 activity, and iron overload, have been widely detected, confirming its direct involvement in neuronal damage (Shen et al., 2023). Increasing evidence suggests that ferroptosis is not only a common feature in the pathological processes of neurological diseases but may also be a key pathogenic link, providing a new perspective for understanding the complex mechanisms of these diseases (Ou et al., 2022). Neurons possess high metabolic activity and intricate synaptic networks, making them physiologically dependent on metal ions such as iron, copper, and zinc. Iron serves as an essential cofactor for the mitochondrial respiratory chain and various neurotransmitter synthesis enzymes; copper is involved in superoxide dismutase (SOD1) activity and neurotransmitter metabolism (Lutsenko et al., 2007); zinc regulates synaptic plasticity and neural signaling (Sensi et al., 2009). Minor disturbances in metal ion homeostasis can lead to increased levels of ROS and accumulation of lipid peroxidation, thereby triggering ferroptosis. Recent studies have shown that ferroptosis not only damages neurons but may also promote Aβ deposition and tau pathology formation in the upstream stages of AD, suggesting that the high demand for metal ions in neurons renders them particularly susceptible to metal-related oxidative stress and ferroptosis.

In AD, ferroptosis interacts closely with Aβ deposition and tau protein neurofibrillary tangles (Zhang et al., 2022). Studies show that there is a significant imbalance in iron metabolism in the brains of AD patients, and this dysregulation of iron homeostasis is considered one of the important factors promoting senile plaque deposition and neurofibrillary tangle formation (Zhang et al., 2022). Iron overload generates a large amount of ROS through the Fenton reaction, directly attacking neuron cell membranes rich in PUFAs and triggering lipid peroxidation (Viktorinova and Durfinova, 2021). Simultaneously, oxidative stress and dysfunction of the endogenous antioxidant system in the AD pathological environment further weaken the ability of neurons to resist lipid peroxidation (Zhang et al., 2022). In particular, GPX4, as a key lipid peroxide repair enzyme, shows significantly decreased activity in AD, leading to a weakened inhibitory effect on ferroptosis (Wu L. et al., 2023). Furthermore, pathological tau protein not only dissociates from microtubules but may also play a complex “double-edged sword” role in the ferroptosis process by affecting mechanisms such as neuronal iron efflux, potentially promoting or, in some cases, delaying cell death (Wu M. et al., 2023). Microglia, as immune cells of the CNS, are abnormally activated in the neuroinflammatory environment of AD. These activated microglia release pro-inflammatory factors and oxidized lipids, exacerbating local oxidative stress and the lipid peroxidation environment, thereby amplifying the ferroptosis process in neurons (Cheng et al., 2021). Therefore, tau protein aggregation and Aβ deposition induce neuronal lipid peroxidation and decreased GPX4 activity, leading to ferroptosis; microglia release pro-inflammatory factors and oxidized lipids to exacerbate this process (Tuo et al., 2026).

In PD, there is progressive loss of dopaminergic neurons in the substantia nigra pars compacta of the midbrain, and ferroptosis is considered one of the key mechanisms leading to this selective neuronal death (Yang et al., 2023). Dopaminergic neurons are exceptionally sensitive to iron-dependent lipid peroxidation damage due to their high metabolic demand, inherent oxidative stress environment, high levels of iron content, and cell membranes rich in PUFAs (Xie C. et al., 2025). Pathological changes closely related to ferroptosis, including abnormal iron metabolism, enhanced lipid peroxidation, and elevated ROS levels, have been observed in the brain tissue of PD patients (Yang et al., 2023). Particularly important is that another core pathological feature of PD, the overexpression of α-synuclein, promotes the occurrence of ferroptosis (Agostini et al., 2023). In Drosophila PD models, the toxic effects of α-synuclein are significantly exacerbated under conditions of reduced intracellular GSH levels or increased iron concentration, while the use of iron chelators or antioxidants can exert protective effects. This directly supports the involvement of ferroptosis in α-synuclein-mediated neurotoxicity (Agostini et al., 2023). This interaction may be bidirectional: α-synuclein aggregation may induce ferroptosis by depleting cellular antioxidant resources or directly interfering with iron metabolism; conversely, lipid peroxidation products and oxidative stress generated during ferroptosis may further promote α-synuclein aggregation and pathological spreading (Erekat, 2025). Additionally, abnormal activation of glial cells in PD can regulate the ferroptosis of dopaminergic neurons by modulating iron homeostasis and lipid peroxidation, constituting a complex regulatory network of neuron-glia interaction in PD ferroptosis (Li et al., 2024a).

Ischemia-reperfusion injury following ischemic stroke is the main driver inducing neuronal ferroptosis, a process that significantly promotes the expansion of the infarct core and the loss of salvageable neurons in the penumbra (Yang et al., 2022). After a stroke, the nerve tissue in the brain region where the ischemic lesion is located (such as the cortex and subcortical structures in the blood supply area of the middle cerebral artery) experiences severe energy metabolism disorder and oxidative stress. During reperfusion, a large amount of oxygen re-enters the ischemic area, generating a burst of ROS through various pathways. Simultaneously, blood-brain barrier disruption leads to erythrocyte extravasation, whose degradation releases a large amount of heme iron. Coupled with dysregulated cerebral iron metabolism regulation post-stroke, this collectively leads to fatal cerebral iron overload (Yang et al., 2022). Excessive accumulation of iron ions catalyzes the conversion of ROS into more destructive hydroxyl radicals via the Fenton reaction, attacking PUFAs on neuronal cell membranes and triggering widespread lipid peroxidation (Liu Y. et al., 2022). This iron-dependent lipid peroxidation process is the core biochemical feature of ferroptosis. Studies confirm that in stroke models, typical markers of ferroptosis, such as GSH depletion, GPX4 activity inhibition, and accumulation of lipid peroxidation products, can be detected (Shen et al., 2023). Inhibiting ferroptosis has been proven in experiments to effectively reduce cerebral infarct volume and improve neurological deficits, which conversely demonstrates the key role of ferroptosis in stroke pathology from an interventional perspective (Yang et al., 2022). Therefore, the “oxidative stress-iron overload-lipid peroxidation” axis triggered by ischemia-reperfusion is the core mechanism for neuronal ferroptosis and the expansion of tissue damage after stroke, making interventions targeting this pathway highly promising (Liu Y. et al., 2022).

## 4 Transcriptional regulation of ferroptosis-related genes by histone lactylation

### 4.1 Lactylation modification activates pro-ferroptosis gene expression

Histone lactylation modification, particularly lactylation at the histone H3K18 site (H3K18-la), has been confirmed as a key epigenetic mechanism regulating the expression of pro-ferroptosis genes. Studies have found that in various disease models, H3K18-la modification is specifically enriched in the promoter regions of multiple pro-ferroptosis-related genes, directly driving their transcriptional activation. For example, in chondrocytes of OA, LDHB-mediated H3K18-la modification directly binds to and activates the promoter of ACSL4, thereby inducing chondrocyte ferroptosis and promoting OA progression (Zhang Y. et al., 2025). Similarly, in nucleus pulposus cells of intervertebral disc degeneration, glycolysis-derived lactate promotes histone H3K18-la, which subsequently upregulates ACSL4 transcription and activates the ferroptosis pathway (Sun K. et al., 2025). Besides ACSL4, other key pro-ferroptosis genes are also regulated by lactylation modification. In a sepsis-induced acute respiratory distress syndrome model, elevated H3K14-la modification in pulmonary endothelial cells is enriched in the promoter regions of TFRC and solute carrier family 40 member 1 (SLC40A1), promoting the expression of these genes and exacerbating endothelial cell ferroptosis (Gong et al., 2020). This evidence indicates that lactylation modification, as a metabolic-epigenetic bridge, can precisely target and activate core pro-ferroptosis genes including ACSL4 and TFRC, driving the initiation of cellular ferroptosis.

The molecular mechanism by which lactylation modification upregulates pro-ferroptosis gene expression primarily involves directly enhancing transcriptional activity by altering chromatin openness or recruiting transcriptional co-activators. Research shows that lactylation modification can remodel local chromatin structure and promote gene transcription. For instance, in a CIRI model, lactate-induced histone H4K12-la in neurons is enriched in the promoter region of the iron transporter-encoding gene Zip14. This modification promotes chromatin accessibility, thereby activating Zip14 transcription and ultimately triggering neuronal ferroptosis (Xu F. et al., 2025). This process clearly demonstrates the mechanism by which lactylation modification directly promotes gene transcription by “opening” chromatin. On the other hand, lactylation modification may also function by recruiting or stabilizing specific transcriptional regulatory complexes. In BRAF inhibitor/MEK inhibitor-resistant melanoma, lactate-induced lactylation modification of lysine-specific demethylase 1 promotes its interaction with Fos-related antigen 1 and enhances the enrichment of Fos-related antigen 1 on the genome, collectively inhibiting TFRC-mediated iron uptake and thereby suppressing ferroptosis (Li et al., 2025). Although this is an example of inhibiting ferroptosis, it conversely confirms that lactylation modification can profoundly affect the ferroptosis-related gene network by regulating the stability of transcription factor complexes. Therefore, lactylation modification can directly act as a “histone code” to alter chromatin conformation or serve as a “protein scaffold” to regulate the assembly and function of the transcriptional machinery, ultimately achieving upregulation of pro-ferroptosis gene expression. Histone lysine lactylation directly modifies lysine residues through lactate, forming metabolism-dependent specific marks. Compared to acetylation, its regulatory pattern is more inclined to respond to metabolic status. Histone lysine lactylation can selectively open chromatin of ischemia or stress-related genes. This specificity may arise from the combined effects of lactate concentration gradients, histone structural accessibility, and recruitment of specific transcriptional complexes.

In the context of neuroinflammation, lactylation-driven changes in gene expression in activated microglia not only enhance their own susceptibility to ferroptosis but may also transmit “death signals” to neighboring neurons via paracrine signaling. Microglia undergo significant glycolytic reprogramming upon activation, producing large amounts of lactate, which provides substrate for histone lactylation modification. Although direct studies on microglia are limited in the existing literature, other inflammation-related cell models provide important clues. For example, in rheumatoid arthritis synovial fibroblasts, H3K18-la upregulates METTL1 and NeuroD1, ultimately enhancing the transcriptional activity of GPX4 to resist ferroptosis (Xu et al., 2026). This suggests that in inflammatory environments, lactylation modification can regulate key antioxidant or pro-death genes, determining cell fate. Extrapolating to activated microglia, the upregulation of ACSL4 expression driven by internal lactylation would promote the production of pro-inflammatory lipid mediators (such as arachidonic acid, etc.). These lipid mediators themselves are key substrates and inducers of ferroptosis and can be released into the extracellular environment. After neighboring neurons take up these PUFA-rich lipid mediators, they become more prone to lipid peroxidation under oxidative stress conditions, inducing neuronal ferroptosis (Deng et al., 2025). This intercellular “death signal” cascade initiated by lactylation and transmitted via lipid mediators may be an important pathological mechanism linking neuroinflammation to neuronal damage, providing a new perspective for understanding neuronal loss in neurological diseases.

### 4.2 Lactylation modification inhibits anti-ferroptosis gene expression

**FIGURE 2:**
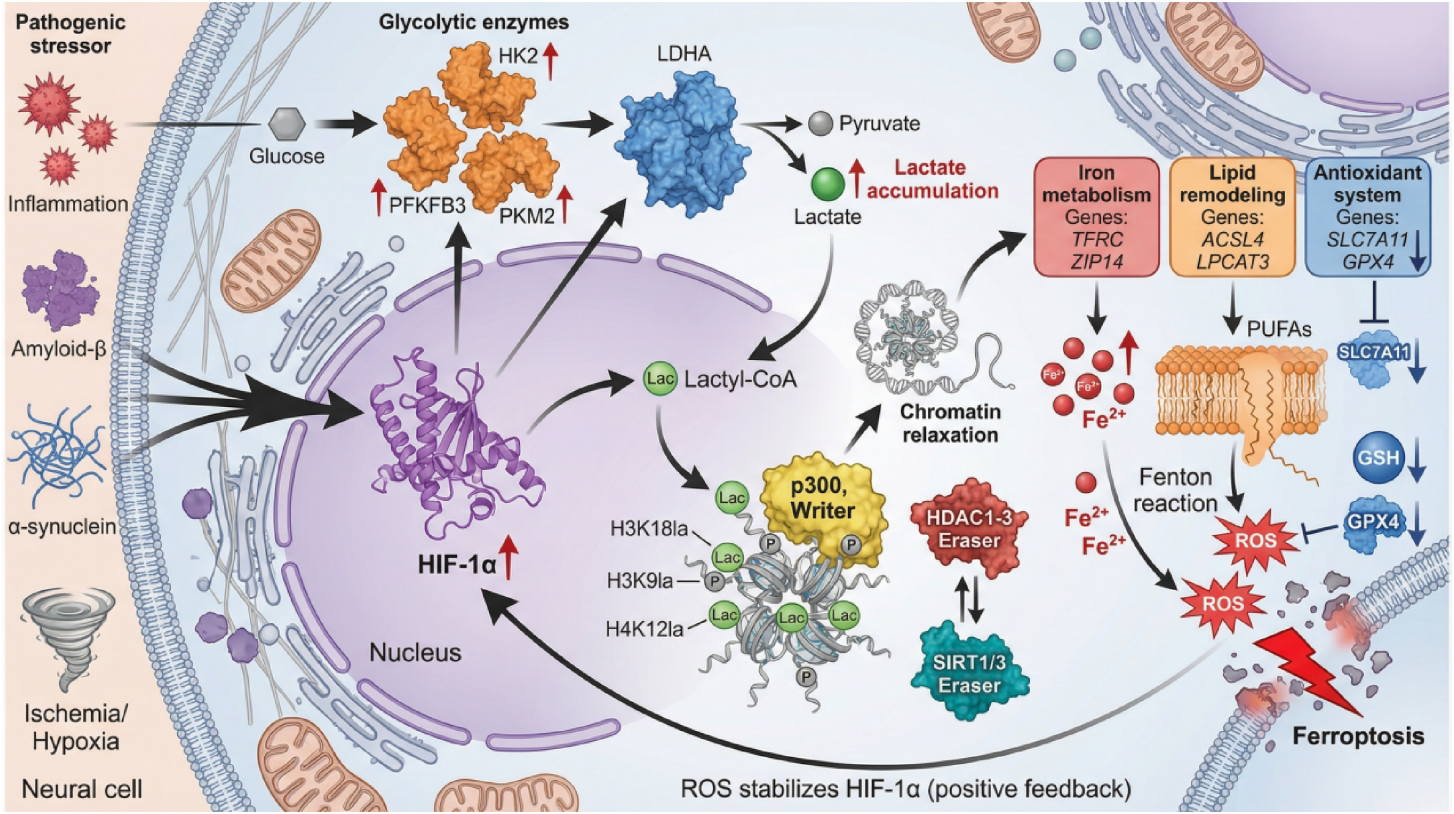
Neurological stressors (inflammation, hypoxia, amyloid-β, α-synuclein, ischemia) stabilize HIF-1α, upregulating glycolytic enzymes (HK2, PFKFB3, PKM2) and promoting lactate production via LDHA. Lactate enters the nucleus, converts to lactyl-CoA, and drives p300-catalyzed histone lactylation (H3K18-la, H3K9-la, H4K12-la; erasers: HDAC1-3, SIRT1/3). Distinct histone lactylation marks occur specifically at the promoter/enhancer loci of ferroptosis-related genes rather than inducing global chromatin opening, triggering targeted chromatin relaxation to selectively modulate three core modules: iron metabolism (TFRC, ZIP14), lipid remodeling (ACSL4, LPCAT3), and antioxidant defense (SLC7A11, GPX4 downregulated). This targeted regulation subsequently induces iron overload, lipid peroxidation, Fenton reactions, and ROS accumulation, ultimately leading to neuronal ferroptosis. A positive feedback loop further amplifies this axis via ferroptosis-derived ROS that further stabilizes HIF-1α.

Histone lactylation modification not only activates pro-ferroptosis genes but may also amplify its promoting effect on the ferroptosis pathway by inhibiting the expression of protective genes through various indirect mechanisms or at different genomic loci (Figure 2). First, a high-lactylation environment may interfere with the activity of deacetylases/delactylases, which are crucial for maintaining the expression of genes in the antioxidant defense system. Deacetylases of the Sirtuin family, such as SIRT1 and SIRT3, play key roles in regulating cellular redox balance (Sun K. et al., 2025). Studies on intervertebral disc degeneration found that elevated lactate levels lead to decreased SIRT3 expression, further promoting ACSL4 lactylation modification and ferroptosis activation (Sun K. et al., 2025). This indicates that lactylation modification may indirectly weaken the cell’s defense against oxidative stress by inhibiting the activity or expression of deacetylases like SIRT3, leading to suppressed expression of antioxidant genes (such as Nrf2 target genes) and creating conditions for ferroptosis occurrence. This interference with delactylase activity constitutes an important aspect of how lactylation modification inhibits cellular protective mechanisms.

Secondly, clear evidence shows that histone lactylation modification can directly inhibit the transcription of key anti-ferroptosis genes. In studies of non-alcoholic steatohepatitis, it was found that irisin TEC upregulates tRF-31R9J, recruits HDAC1, thereby reducing histone lactylation and acetylation levels in the promoter regions of pro-ferroptosis genes ATF3, ATF4, and CHAC1, and subsequently inhibiting their expression (Zhu et al., 2025). This mechanism reveals the direct role of lactylation modification in gene suppression. More importantly, studies suggest that lactylation modification may inhibit the transcription of SLC7A11, a key subunit of the cystine/glutamate antiporter System Xc-. System Xc- is responsible for transporting extracellular cystine into the cell, which is the rate-limiting step for GSH synthesis. GSH is a necessary cofactor for GPX4 to exert its antioxidant function. Although the provided references do not directly report the inhibition of SLC7A11 by lactylation, multiple studies support the logic that lactylation modification broadly regulates the ferroptosis-related gene network. For example, in ovarian cancer, H3K18-la modification activates TRA2A transcription, which then regulates the alternative splicing of STIL, ultimately inhibiting ferroptosis (Gao et al., 2025). These studies collectively indicate that lactylation modification can weaken the cell’s ability to synthesize GSH and combat lipid peroxidation by activating or inhibiting downstream target genes.

In summary, histone lactylation modification exhibits a “dual regulatory” mode on the ferroptosis pathway: on one hand, it “pushes” the ferroptosis process by promoting the expression of pro-ferroptosis genes like ACSL4. On the other hand, it “disarms” cellular protective mechanisms by inhibiting the activity of deacetylases like SIRT3 or directly inhibiting the transcription of anti-ferroptosis-related genes. In pathological environments with hyperactive glycolysis, the massive accumulation of lactate drives widespread histone lactylation. This dual regulatory mode makes cells more susceptible to ferroptosis or, conversely, enables them to acquire resistance by specifically activating certain anti-ferroptosis pathways. Therefore, histone lactylation modification becomes a key epigenetic switch connecting cellular metabolic status and ferroptosis.

## 5 Research on the glycolysis-lactylation-ferroptosis axis in AD

### 5.1 Metabolic reprogramming and lactylation modification landscape in the AD brain

**FIGURE 3:**
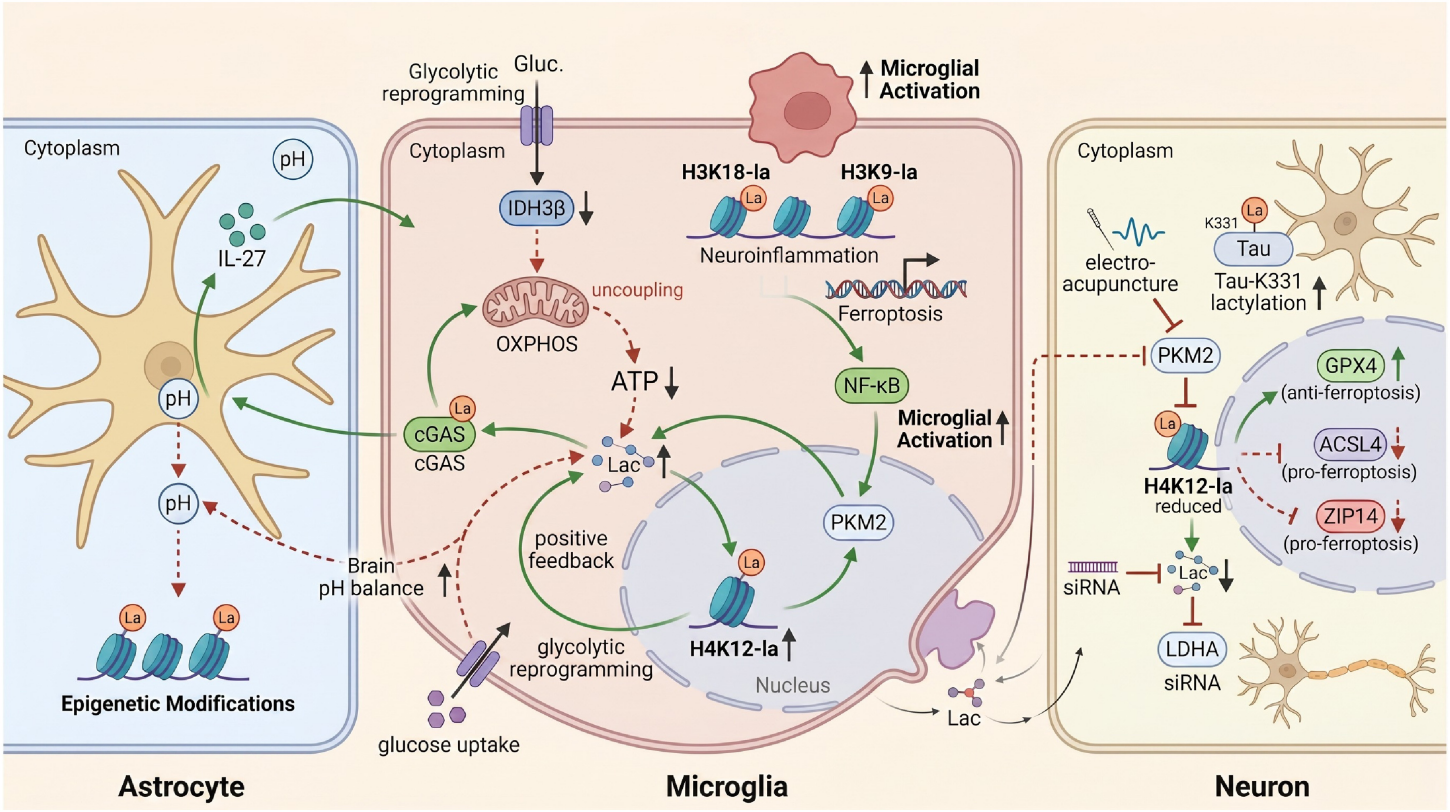
In AD brains, down regulated IDH 3β impairs tricarboxylic acid cycle and OXPHOS, triggering metabolic reprogramming, energy deficiency, and lactate accumulation. Excess lactate drives lactylation modifications: tau K331 lactylation exacerbates AD-like pathology; histone lactylation (H3K18-la, H3K9-la, H4K12-la) remodels gene transcription involved in neuro inflammation and ferroptosis. Microglia exhibit a glycolysis/H4K12-la/PKM2 positive feedback loop, promoting pro-inflammatory activation via NF-κB signaling. Astrocyte dysfunction disrupts brain pH homeostasis and modulates microglial metabolic reprogramming through cGAS lactylation. Collectively, the glycolysis-lactylation-ferroptosis axis drives AD pathogenesis via metabolic, epigenetic, and inflammatory crosstalk.

Significant metabolic reprogramming exists in the brains of AD patients (Figure 3), characterized by abnormal activation of the glycolytic pathway and elevated lactate levels. Positron emission tomography studies have confirmed regional abnormalities in glucose metabolism in the brains of AD patients, and proteomic analysis of postmortem brain tissue has further revealed upregulation of glycolytic enzyme expression and increased lactate levels (Hai et al., 2025). This metabolic shift, i.e., the deviation from OXPHOS to aerobic glycolysis, appears in the early stages of AD and is considered a key driver of disease progression (Gu et al., 2026b). In AD transgenic mice, regional decreases in brain tissue pH and increased levels of histone H4K12-la in neurons can be observed even before the onset of cognitive decline, directly supporting the association between lactate accumulation and metabolic reprogramming (Yao et al., 2025). In-depth studies indicate that this metabolic dysregulation may originate from the downregulation of isocitrate dehydrogenase 3β, a key enzyme in the tricarboxylic acid cycle. Its knockdown leads to OXPHOS uncoupling, reduced energy metabolism, and lactate accumulation, thereby forming a positive feedback inhibitory loop that promotes AD-like pathology (Wang X. et al., 2024). Furthermore, lactylation modification of tau protein in the brains of AD patients, particularly at the K331 site, has also been found to be significantly elevated, directly linking metabolic dysregulation to tauopathy (Zhang X. et al., 2025). These findings collectively depict a metabolic remodeling landscape in the AD brain characterized by enhanced glycolysis and increased lactate production.

Chromatin immunoprecipitation sequencing data from AD model brain tissues or cells show that histone lactylation modifications, particularly H3K18-la and H3K9-la, exhibit specific enrichment at certain gene loci involved in key pathological processes such as neuroinflammation and ferroptosis (Cao M. et al., 2025). For example, in the pathological context of AD, H4K12-la was found to be enriched in the promoter regions of glycolysis-related genes in microglia, enhancing glycolytic activity by activating transcription and forming a “glycolysis/H4K12-la/PKM2” positive feedback loop that drives the pro-inflammatory phenotypic activation of microglia (Pan et al., 2022). This loop has been confirmed in brain samples from both AD patients and 5XFAD mice, with significantly elevated H4K12-la levels in microglia adjacent to Aβ plaques (Pan et al., 2022). H3K18-la has also been reported to potentially enhance microglial activation by activating the nuclear factor kappa B (NF-κB) pathway (Tang et al., 2025). These epigenetic modifications reshape the chromatin landscape, leading to abnormal expression of genes involved in neuroinflammation and ferroptosis-related genes (Cao M. et al., 2025). Therefore, lactylation modification, as a metabolic-epigenetic bridge, profoundly influences the neuroinflammatory microenvironment and neuronal damage process in AD by specifically regulating the expression of pro-inflammatory and pro-ferroptosis genes.

The “reactive” transformation of astrocytes and microglia is the primary cellular source of lactate and lactylation modifications in the AD brain. These modifications reshape the epigenetic landscape of glial cells, polarizing them toward pro-inflammatory and neurotoxic phenotypes (Wang et al., 2026). Under AD pathological stimulation, microglia undergo significant glycolytic reprogramming. The lactate they produce not only serves as an energy substrate but also acts as a signaling molecule driving histone lactylation and altering gene expression (Wang et al., 2026). This epigenetic remodeling mediated by the glycolysis-histone lactylation axis is the core mechanism driving the polarization of microglia toward a pro-inflammatory phenotype. Astrocytes also undergo metabolic and functional changes in AD. They respond to pathological signals by secreting factors such as interleukin-27, which in turn regulates microglial reprogramming by modulating the lactylation of cyclic GMP-AMP synthase (Du et al., 2026). Furthermore, astrocytes play a key role in maintaining brain pH homeostasis, with lactate levels being a major determinant of brain pH (Nohesara et al., 2024). The pH imbalance caused by lactate metabolism disorder in AD may further affect various epigenetic modifications, including histone lactylation, thereby forming a complex pathological network in glial cells and neurons. These reactive glial cells, by producing lactate and mediating lactylation modifications, collectively shape the pro-inflammatory, pro-injury microenvironment in the AD brain, highlighting the great potential of targeting the glial cell metabolic-epigenetic axis as a new therapeutic strategy for AD.

### 5.2 Neuron-glia dialogue mediated by this axis and research prospects

Given the central role of the glycolysis-histone lactylation axis in driving neuroinflammation and neuronal ferroptosis, targeting key nodes of this axis has become a therapeutic strategy. In animal models of neurodegenerative diseases such as AD, inhibiting key glycolytic enzymes (e.g., PKM2) or histone lactylation “writer” enzymes in microglia has shown significant therapeutic effects (Jia et al., 2026; Li J. et al., 2026). Electroacupuncture treatment can effectively reduce H4K12-la levels in neurons by inhibiting PKM2 (Xu F. et al., 2025). Similarly, genetically silencing LDHA can reduce lactate production, thereby lowering overall histone lactylation levels in the brain (Zhu et al., 2026). These interventions can effectively alleviate neuroinflammatory responses, with mechanisms possibly including inhibiting the release of pro-inflammatory factors and the polarization of macrophages/microglia toward a pro-inflammatory phenotype. More importantly, by cutting off upstream lactylation signals, the expression of anti-ferroptosis genes (e.g., GPX4) in neurons can be restored or the transcription of pro-ferroptosis genes (e.g., ACSL4, ZIP14) can be inhibited, thereby effectively suppressing neuronal ferroptosis (Sun K. et al., 2025; Xu F. et al., 2025). Ultimately, these molecular and cellular improvements translate into behavioral benefits, manifested as significant cognitive enhancement in animal models (Qin et al., 2025b). This indicates that precise intervention in the glycolysis-lactylation axis provides a potential pathway for treating neurological diseases characterized by neuroinflammation and ferroptosis.

## 6 Research on the glycolysis-lactylation-ferroptosis axis in PD

### 6.1 Unique vulnerability of substantia nigra dopaminergic neurons

**FIGURE 4:**
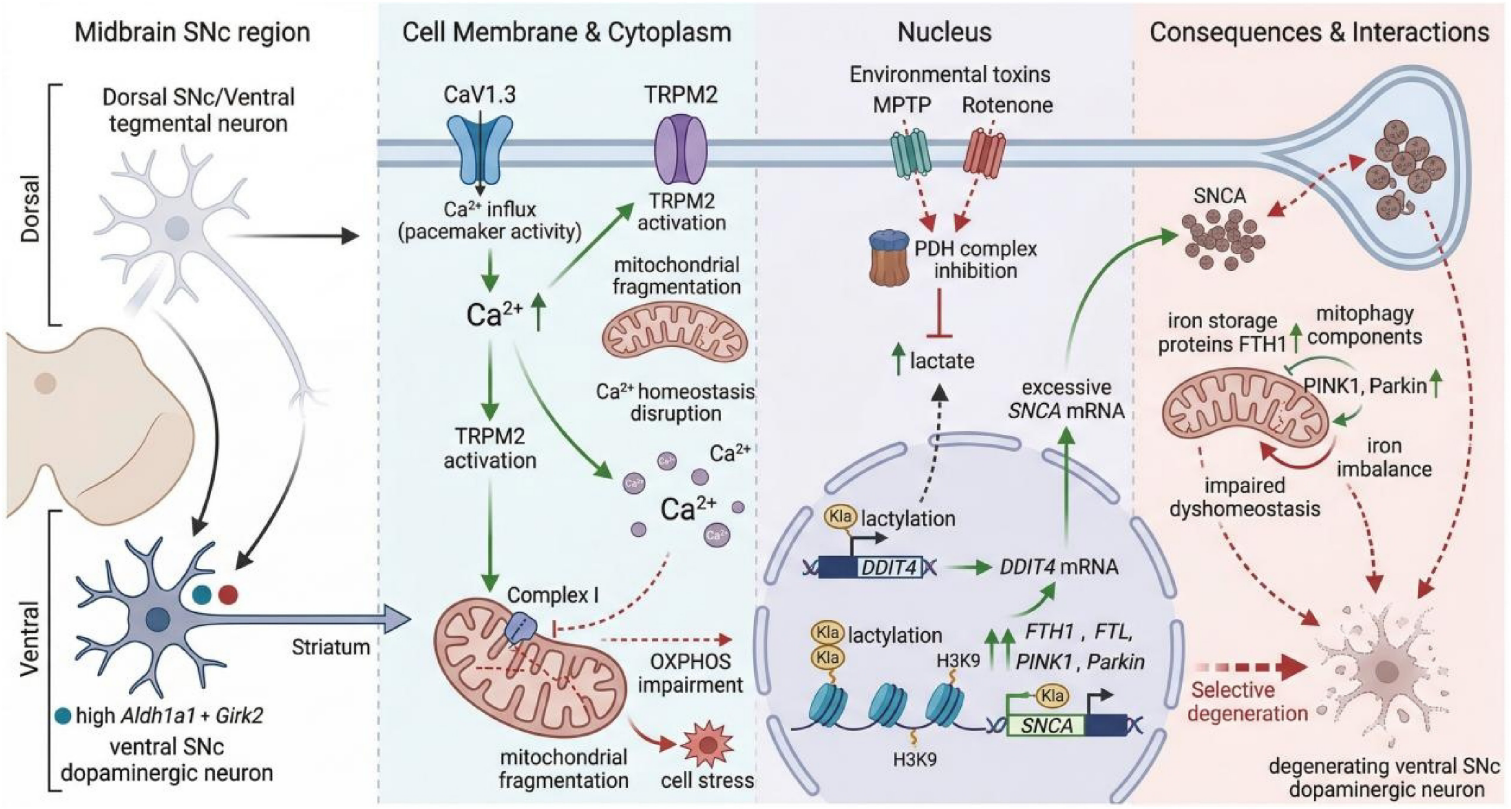
In PD, dopaminergic neurons in the SNc degenerate, especially ventral-tier neurons expressing Aldh1a1 and Girk2. Their pacemaker activity relies on CaV1.3-mediated Ca^2+^ influx, increasing vulnerability. PD patient-derived neurons show high TRPM2 expression, disrupting mitochondrial dynamics and calcium homeostasis. SNCA dysregulation is a core PD pathology. Neurotoxins (MPTP, rotenone) inhibit pyruvate dehydrogenase, elevating lactate and histone lactylation. This enhances DDIT4 and SNCA transcription (e.g., H3K9-la), promoting dopaminergic neuron death. Lactylation also modulates iron storage genes (FTH1, FTL) and mitophagy genes (PINK1, Parkin). Thus, lactylation links mitochondrial dysfunction, iron imbalance, and key pathogenic pathways in PD progression.

Dopaminergic neurons in the substantia nigra pars compacta (SNc) exhibit significant and selective degeneration in PD (Figure 4). These neurons have an extremely high basal metabolic rate, requiring continuous production of large amounts of ATP to maintain their autonomous pacemaking activity and unique morphological features, making them particularly sensitive to impairments in OXPHOS or dysfunction of mitochondrial electron transport chain complex I (Ni and Ernst, 2022). This high metabolic activity is one of the key reasons for their susceptibility to oxidative stress. Furthermore, the pacemaking activity of SNc dopaminergic neurons, especially those in the ventral tier, is accompanied by calcium influx through L-type calcium channels CaV1.3, which increases intracellular calcium concentration and may exacerbate the cell death process (Verma and Ravindranath, 2019). In PD mouse models, the mRNA levels of a specific splice variant of the CaV1.3 channel, CaV1.342A, are higher in the SNc, which may further amplify calcium-dependent damage (Verma and Ravindranath, 2019). The selective vulnerability of SNc neurons is also reflected in differences in their molecular phenotypes. In primate models, dopaminergic neurons located in the ventral tier of the SNc, which are rich in Aldh1a1 and Girk2, are vulnerable and primarily project to the striatum; whereas neurons located in the dorsal tier and ventral tegmental area, marked by calcium-binding proteins, are relatively resilient (Del Rey et al., 2024). This distribution of molecular phenotypes closely aligns with the dorsoventral axis susceptibility of neuronal degeneration. Proteomic analysis further reveals molecular differences between dorsal and ventral tier neurons in the SNc, involving variations in the expression of proteins related to the cytoskeleton, neuronal plasticity, and calcium homeostasis, providing deeper insights into understanding selective vulnerability and protective mechanisms targeting specific neuronal subpopulations (Steinbach et al., 2024).

In the pathological progression of PD, the vulnerability of SNc dopaminergic neurons is closely related to changes in their microenvironment. Neuroinflammation and immune cell infiltration are key features of PD neurodegeneration (Balzano et al., 2024). Studies in non-human primate PD models have found that the ventral region of the SNc is typically more vascularized than other regions, potentially making this area more susceptible to immune cell infiltration and exacerbating the vulnerability of dopaminergic neurons (Balzano et al., 2024). Additionally, the activation of glial cells, particularly microglia and astrocytes, plays an important role in the pathogenesis of PD. Exposure to environmental toxins, such as λ-cyhalothrin, induces neuroinflammation in the SNc and striatum, manifested by upregulation of pro-inflammatory cytokines, downregulation of anti-inflammatory cytokines, and an increase in glial cell markers (GFAP, Iba-1), leading to dopaminergic neuron damage through activation of the NF-κB and JAK/STAT signaling pathways (Kumari et al., 2023). Vulnerable dopaminergic neurons are particularly sensitive to mitochondrial dysfunction (De Miranda et al., 2020). Loss of Parkin function leads to persistently elevated mitochondrial hydrogen peroxide levels and disrupted GSH redox balance in specific dopaminergic neuron clusters in Drosophila, triggering selective degeneration (Houlihan et al., 2022). In dopaminergic neurons differentiated from induced pluripotent stem cells derived from PD patients, preferential expression of the TRPM2 channel is associated with neuronal vulnerability, and its activation can lead to altered mitochondrial dynamics and disrupted calcium homeostasis, thereby mediating selective death (Ye et al., 2023). The interaction between intrinsic characteristics and external stressors collectively determines the fate of SNc dopaminergic neurons in PD.

### 6.2 Regulatory network of lactylation on PD-related genes and potential strategies

Research suggests that lactylation modification may directly regulate genes associated with PD pathogenesis. For example, abnormal expression of the α-synuclein gene (SNCA) is one of the core pathological features of PD. Studies indicate that environmental neurotoxins MPTP and rotenone can inhibit pyruvate dehydrogenase complex activity, increase lactate production, and subsequently drive histone lactylation in the promoter region of the DDIT4 gene, leading to upregulation of its expression and ultimately promoting dopaminergic neuron death (Hong et al., 2026). This high-lactate environment may enhance histone lactylation modifications, particularly lactylation at the H3K9 site, thereby promoting excessive transcription of genes such as SNCA (Moreno-Yruela et al., 2022b). This lactate-driven epigenetic regulation directly links glycolytic metabolic reprogramming with the expression of key pathological proteins in PD, providing a possible explanation for the abnormal aggregation of SNCA in PD (Cao M. et al., 2025). Histone lactylation modifications may also broadly regulate gene networks involved in iron homeostasis and mitophagy, and dysfunction in these pathways is associated with increased susceptibility to ferroptosis. In the pathological progression of PD, mitochondrial dysfunction and iron metabolism imbalance are key factors leading to dopaminergic neuron death (Tu et al., 2025). Studies show that lactylation modification can target the expression of genes related to iron storage proteins such as ferritin heavy chain 1 (FTH1) and ferritin light chain (FTL), as well as core mitophagy genes like PTEN-induced kinase 1 (PINK1) and Parkin (Lei et al., 2026). These findings collectively outline a complex regulatory network mediated by lactylation, connecting metabolic abnormalities, epigenetic changes, mitochondrial dysfunction, and susceptibility to ferroptosis.

Given the important role of lactylation in PD gene regulation, targeting lactate metabolism or the lactylation modification process has become a potential therapeutic strategy. Using LDH inhibitors to reduce lactate production, or using delactylation enzyme activators (such as SRT2104), has shown potential in PD cell and animal models to protect dopaminergic neurons and alleviate motor symptoms. The core of these interventions lies in interrupting the pathological axis of “enhanced glycolysis—lactate accumulation—histone lactylation—pathogenic gene expression” (Gu et al., 2026a). Mendelian randomization studies provide genetic evidence for targeting lactylation-related genes; for example, increased expression of the SIRT1 gene shows a protective effect against PD dementia (Zhang H. et al., 2025). These findings collectively indicate that modulating lactate production or lactylation modification levels may restore normal gene expression profiles, reduce neuroinflammation and ferroptosis, thereby providing direction for PD treatment.

## 7 Research in ischemic stroke and reperfusion injury

### 7.1 Acute metabolic changes in the brain post-stroke and the lactate storm

**FIGURE 5:**
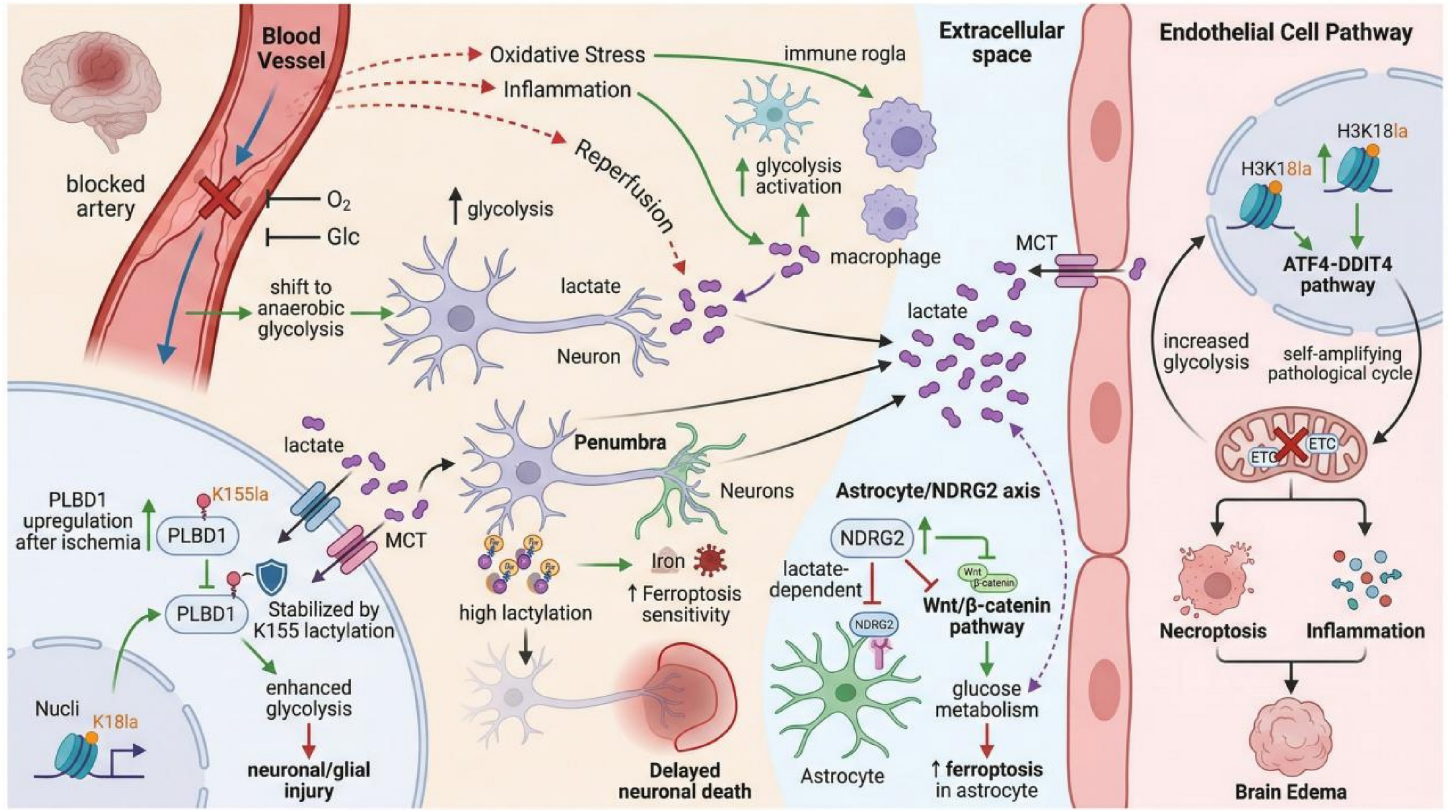
Cerebral ischemia causes oxygen and glucose deprivation, triggering anaerobic glycolysis and rapid lactate accumulation. Reperfusion aggravates oxidative stress and inflammation, sustaining persistent lactylation in the penumbra. This hyper-lactylated environment increases neuronal and glial sensitivity to ferroptosis, leading to delayed neuronal death and infarct expansion. Ischemia-upregulated phospholipase B domain-containing protein 1 is stabilized by lysine-155 lactylation, enhancing glycolysis and injury. In astrocytes, elevated N-myc downstream-regulated gene 2 inhibits Wnt/β-catenin signaling to promote ferroptosis. In endothelial cells, lactate-induced H3K18-la activates the ATF4-DDIT4 pathway, forming a glycolysis–lactylation pathological cycle. Extracellular lactate uptake via monocarboxylate transporters further drives histone lactylation.

Cerebral ischemia disrupts the supply of oxygen and glucose (Figure 5), forcing cells to undergo anaerobic glycolysis, which rapidly produces and accumulates large amounts of lactate, leading to tissue acidosis and a “lactate storm” (Zhang R. et al., 2025). Advanced techniques such as magnetic resonance spectroscopy imaging have confirmed that in patients with acute ischemic stroke, lactate signals are significantly elevated in the infarct core and surrounding areas, and regions that ultimately progress to infarction exhibit markedly higher lactate levels compared to areas capable of recovery (Li et al., 2020). This pathological lactate accumulation not only reflects disturbances in energy metabolism but also serves as a crucial energy substrate and signaling molecule, playing a dual role in the post-stroke pathophysiological process (Cai et al., 2022). Studies have shown that exogenous administration of L-lactate in rodent stroke models can reduce lesion volume and improve neurological outcomes, suggesting that lactate may have neuroprotective effects under specific conditions (Hyacinthe et al., 2020). However, this protective effect is highly dependent on the dynamic balance of lactate metabolism. Real-time metabolic tracking using hyperpolarized 13C-labeled lactate or pyruvate has revealed significant alterations in the metabolism of both substrates in the mouse brain post-stroke, with their metabolic ratios and kinetic rates progressively declining within 1–2 h after reperfusion, reflecting the metabolic reprogramming initiated during the acute phase to meet energy demands (Lê et al., 2025).

Although reperfusion restores oxygen supply, it can exacerbate oxidative stress and trigger inflammatory responses, persistently activating glycolysis in immune cells, thereby maintaining high levels of lactylation modification in the peri-infarct area (penumbra) for an extended period (Wei et al., 2025). The hypoxia-inducible factor (HIF) pathway plays a central regulatory role in this process. Activating HIF through genetic or pharmacological means (e.g., roxadustat) can induce metabolic reprogramming in brain-resident cells, including neurons and astrocytes, enhancing glycolytic capacity and lactate production, thereby increasing cellular tolerance to ischemic stress (Madai et al., 2024). However, this metabolic shift is not always beneficial. In neurons, excessive HIF-1 activation may redirect glucose-6-phosphate from the NADPH-producing pentose phosphate pathway to the glycolytic pathway by upregulating 6-phosphofructo-2-kinase/fructose-2,6-bisphosphatase-3, thereby reducing the intrinsic antioxidant reserves of neurons and increasing their susceptibility to ischemic stress (Madai et al., 2024). Furthermore, persistent hyperglycolysis and lactate production post-reperfusion intertwine with oxidative stress and neuroinflammation, collectively shaping the pathological microenvironment of the penumbra.

This pathological hyperlactylation environment, through the aforementioned mechanisms, significantly upregulates the susceptibility of neurons and glial cells in the penumbra to ferroptosis, leading to delayed neuronal death and infarct expansion (Wu et al., 2024). Lactate is not only a product of glycolysis; its accumulation can also drive protein lactylation modification, a newly discovered post-translational modification that plays a key role in stroke pathology. For example, phospholipase B domain-containing protein 1 is upregulated after ischemia, and lactylation modification at lysine 155 enhances its stability, thereby exacerbating glycolysis and promoting brain injury (Zhou et al., 2025). In astrocytes, upregulation of N-myc downstream-regulated gene 2 expression inhibits glucose metabolism and promotes ferroptosis by suppressing the Wnt/β-catenin signaling pathway, a process closely linked to alterations in lactate metabolism (Wu et al., 2024). Lactate accumulation may also affect gene expression through epigenetic mechanisms such as histone lactylation. In endothelial cells, lactate accumulation increases H3K18-la, subsequently activating the ATF4-DDIT4 pathway, leading to mitochondrial dysfunction and impaired electron transport chain activity, forming a self-amplifying pathological cycle of glycolysis/H3K18-la/ATF4-DDIT4, ultimately promoting necroptosis and inflammation and exacerbating cerebral edema (Wang Y. et al., 2024). Based on these findings, the “lactate storm” after stroke and the lactylation it drives may serve as a potential regulatory axis linking metabolic crisis, epigenetic regulation, and ferroptosis, providing important clues for understanding the mechanisms of infarction progression and neurological deficits.

### 7.2 Targeting this axis as a neuroprotective strategy for stroke and research prospects

Animal experiments provide direct evidence for targeting the glycolysis-ferroptosis axis. Following the onset of ischemic stroke, acute compensatory glycolysis is strongly activated, leading to massive lactate production. The sharp increase in extracellular lactate levels is taken up by cells such as neurons via monocarboxylate transporters, potentially driving histone lactylation modifications and altering gene expression profiles, thereby exacerbating the metabolic crisis. Therefore, administering monocarboxylate transporter inhibitors early during reperfusion, aimed at reducing neuronal uptake of extracellular lactate, can theoretically lower the intracellular lactate pool and downregulate pathological histone lactylation levels. The core of this intervention strategy lies in interrupting lactate-mediated epigenetic signaling, potentially alleviating ferroptosis and subsequent brain injury driven by metabolic reprogramming from the upstream. Thus, targeting lactate transport offers an early therapeutic window.

Developing drugs that directly regulate histone lactylation modifications is another promising neuroprotective approach. Strategies aimed at directly reversing this abnormal modification have emerged. Using selective p300 inhibitors (e.g., C646) can inhibit the activity of histone lactyltransferases, thereby preventing lactylation modifications. On the other hand, activating histone delactylases can promote the removal of existing lactylation marks. In stroke models, such interventions directly targeting epigenetic modifications demonstrate broader neuroprotective effects compared to traditional antioxidants. Their advantage lies in the ability to more precisely intervene in the core disease-driving mechanisms rather than merely counteracting downstream oxidative damage consequences. The metabolic reprogramming framework redefines stroke as a modifiable metabolic disease, and treatments targeting its epigenetic level represent an important exploratory direction within this new paradigm.

Given the complexity of the glycolysis-ferroptosis axis, combination therapy strategies are being explored to achieve synergistic protective effects and salvage neurons with reversible damage within the ischemic penumbra. A single intervention point may be insufficient to address the pathological process involving multiple steps and cell types. For example, combining ferroptosis-specific inhibitors (e.g., Ferrostatin-1) with glycolysis modulators can theoretically simultaneously block the terminal event of the injury pathway (iron-dependent lipid peroxidation) and the upstream drivers (excessive glycolysis activation and subsequent lactate accumulation). This combination strategy can more comprehensively cover different stages of the metabolic crisis: the acute glycolytic-excitotoxic crisis and the subacute lipid peroxidation cascade (Wang et al., 2025b). This suggests that combined regulation of metabolic and supportive functions in glial cells while protecting neurons from ferroptosis in stroke may yield synergistic effects. Decoding cell type-specific vulnerabilities through spatiotemporal metabolomics and single-cell omics, such as astrocyte lipid droplets, microglial succinate accumulation, and the neuron-glial lactate shuttle, provides potential target combinations for designing precise combination therapies.

## 8 Research in ALS and other neurological diseases

### 8.1 Metabolic abnormalities and oxidative stress in ALS

ALS is a fatal neurodegenerative disease. Metabolic dysfunction emerges very early in the pathogenesis of ALS, even before symptom onset, and its pathological process is closely associated with significant metabolic dysfunction and oxidative stress (Burg and Van Den Bosch, 2023). In ALS patients and model animals such as those with SOD1 mutations, significant bioenergetic metabolic dysfunction and oxidative damage are present in the spinal cord and motor cortex (Tefera et al., 2021). This metabolic abnormality is not only evident at the CNS level but also manifests as systemic metabolic disturbances, such as hypermetabolism, weight loss, and dyslipidemias, which are closely related to patient survival (De Silva et al., 2022). Metabolomic and proteomic analyses have further revealed dysregulation of key metabolic pathways including purine metabolism, methionine cycle, and glycolysis in ALS mouse models (Xu et al., 2023). Concurrently, oxidative stress is a recognized core pathological mechanism in ALS, which induces inflammatory responses, exacerbates mitochondrial dysfunction, and directly leads to motor neuron injury and death (Park and Yang, 2021). Elevated levels of oxidative stress markers and a weakened antioxidant defense system have been observed in both ALS patients and animal models (Beers et al., 2022). This oxidative damage is intertwined with metabolic abnormalities. Defects in glucose metabolism within the CNS lead to reduced mitochondrial energy production and excessive ROS generation, collectively driving disease progression (Tefera et al., 2021).

The abnormal activation of astrocytes and microglia is a key driver of ALS disease progression (Wu et al., 2025). These activated glial cells exhibit significant metabolic reprogramming, particularly hyperactive glycolysis (Esteve et al., 2026). This shift in metabolic state may drive their transformation into a neurotoxic phenotype through the production of excessive lactate. When activated astrocytes face metabolic stress such as fatty acid overload, they accumulate lipid droplets accompanied by an NF-κB-driven pro-inflammatory response. This phenotypic shift is associated with toxic effects on motor neurons (Esteve et al., 2026). Neurotoxic glial cells release toxic substances such as ROS and pro-inflammatory factors, inducing ferroptosis in motor neurons (Russo et al., 2025). Ferroptosis is an iron-dependent, regulated cell death characterized by lipid peroxidation. Its key features include oxidative stress, GSH depletion, and mitochondrial dysfunction, which are precisely common phenomena in ALS pathology (Wang L. Y. et al., 2024). Studies have shown that in ALS cell models carrying FUS mutations, cells exhibit increased susceptibility to ferroptosis, manifested as exacerbated lipid peroxidation and an imbalanced antioxidant system (Ismail et al., 2024). Analyses of blood and post-mortem tissues from ALS patients have also revealed alterations in gene expression related to the ferroptosis pathway (Zhang Q. et al., 2022). Therefore, activated glial cells may promote an oxidative environment and release toxic signals through hyperactive glycolysis and lactate production, triggering the ferroptosis program in motor neurons.

Research on ALS models related to TDP-43 or FUS proteinopathies has found significant intersections between the abnormal aggregation of these RNA-binding proteins, cellular metabolic dysregulation, and the ferroptosis pathway, with histone lactylation potentially involved. The abnormal aggregation of TDP-43 triggers oxidative stress, leading to global mitochondrial imbalance, and mitochondrial dysfunction and oxidative stress are core drivers of ferroptosis (Zuo et al., 2021). Similarly, FUS mutations are also associated with mitochondrial dysfunction and oxidative damage, increasing cellular vulnerability to ferroptosis. The pathological aggregation of these RNA-binding proteins disrupts normal RNA metabolism, which may in turn affect the gene expression of various metabolic pathways, including glycolysis (Tsuiji, 2025). Abnormal hyperactivity of glycolysis leads to massive lactate accumulation. lactylation modification may drive the transformation of glial cells into a pro-inflammatory, neurotoxic phenotype. In neurons, abnormal metabolic states and lactylation may directly promote the occurrence of ferroptosis by regulating genes related to iron metabolism, antioxidant defense, and lipid peroxidation (Liao et al., 2025; Xiang et al., 2024). Therefore, in the context of TDP-43 or FUS proteinopathies, lactate produced by glycolysis may, through lactylation modification, link upstream protein aggregation and RNA metabolic disturbances with downstream oxidative stress, mitochondrial dysfunction, and ferroptosis, driving ALS neurodegeneration.

### 8.2 Research in multiple sclerosis (MS) and traumatic brain injury

In MS, macrophages and microglia within the CNS are highly dependent on glycolysis to meet the energy demands of their rapid activation and proliferation. Enhanced glycolysis also leads to significant lactate accumulation. A study on MS found that the inflammatory cytokine interferon-γ can induce the expression of the immunoproteasome subunit PSMB8 in neurons, disrupting proteasome balance and leading to reduced activity (Woo et al., 2025). This dysfunction caused the accumulation of the key metabolic regulator PFKFB3, which in turn enhanced neuronal glycolysis while reducing the activity of the pentose phosphate pathway, ultimately leading to oxidative damage and the occurrence of ferroptosis (Woo et al., 2025). This finding links neuroinflammation, metabolic disturbances, and a specific form of cell death in the MS pathological process. Oligodendrocytes are responsible for forming and maintaining myelin. Their cell membranes are rich in lipids and are highly sensitive to lipid peroxidation and ferroptosis. Therefore, the glycolysis-lactylation axis driven by activated immune cells may directly damage oligodendrocytes by promoting oxidative stress and ferroptosis, hindering myelin regeneration, and thus contributing to the chronic inflammation and neurodegeneration in MS.

In traumatic brain injury, the complex secondary injury cascade that occurs after the primary mechanical insult is the main cause of neurological deterioration. This process involves inflammation, metabolic crisis, and the activation of various forms of regulated cell death. Preliminary evidence suggests that ferroptosis is involved in neuronal damage following traumatic brain injury (Long et al., 2025). Bioinformatic analysis found an intersection between ferroptosis-related genes and the glycolysis/gluconeogenesis gene set in traumatic brain injury, suggesting a potential link between the two (Wang et al., 2025d). Further analysis identified the HIF-1 signaling pathway as a key pathway for ferroptosis after traumatic brain injury and screened hub genes including PGK1, PKM, and HIF-1α (Wang et al., 2025d). In animal models, the injury core presents a high-lactate environment, suggesting that the glycolysis-lactylation axis may be an important upstream regulatory mechanism (Xu L. et al., 2025). Following traumatic brain injury, the level of histone lactylation modification in neurons increases, and exogenous lactate treatment can alleviate a composite programmed neuronal death by upregulating the expression of PSMD14 protein (Xu L. et al., 2025). PSMD14 stabilizes PKM2 protein through deubiquitination, thereby activating PINK1-mediated mitophagy, maintaining mitochondrial homeostasis, reducing ROS production, and ultimately inhibiting neuronal death (Xu L. et al., 2025). This mechanism links the post-injury high-lactate environment, histone lactylation modification, metabolic enzyme stabilization, mitochondrial quality control, and cell death inhibition. Therefore, metabolic reprogramming after traumatic brain injury, particularly the high lactate produced by hyperactive glycolysis, may broadly affect downstream cell death pathways including ferroptosis through the induction of protein lactylation modifications, becoming a key node connecting metabolic dysregulation and cell fate. Targeting this regulatory axis may provide therapeutic strategies for improving traumatic brain injury prognosis.

## 9 Research methods and technological advances

### 9.1 Detection and quantification techniques for histone lysine lactylation

Histone lysine lactylation, as an emerging epigenetic modification, requires precise detection and quantification as the foundation for a deeper understanding of its role in physiological and pathological processes. Mass spectrometry-based proteomics technology is currently the gold standard method for discovering and globally quantifying histone lactylation modifications (Zhao W. et al., 2025). This method does not rely on specific antibodies and enables unbiased, high-throughput analysis of histone extracts. By optimizing a series of strategies including histone extraction, enzymatic digestion, enrichment, and mass spectrometry detection, mass spectrometry technology cannot only comprehensively identify known histone modifications, such as acetylation and methylation, but also greatly facilitate the discovery of novel modifications including lactylation, succinylation, and crotonylation (Zhao W. et al., 2025). This allows researchers to precisely identify the specific lysine sites where lactylation occurs and perform quantitative analysis of their relative abundance, thereby mapping complex histone modification landscapes and profoundly revealing the complexity of the histone code and its underlying epigenetic regulatory mechanisms. However, mass spectrometry typically requires relatively large starting sample amounts and places high demands on instrumentation and operational expertise.

To study the function of histone lactylation in specific biological contexts, site-specific antibodies have become indispensable tools (Jian et al., 2026). For example, antibodies targeting H3K18-la or H3K14-la are widely used in Western Blot to detect overall level changes of specific lactylation modifications, or in immunofluorescence techniques to observe their subcellular localization (Chen W. et al., 2026). More importantly, chromatin immunoprecipitation techniques combined with specific antibodies enable researchers to investigate the distribution of specific histone lactylation modifications across the genome, thereby directly linking epigenetic marks to gene transcription regulation (Jian et al., 2026). A study on diabetic keratopathy precisely utilized CUT&Tag technology combined with an H3K14-la antibody to achieve high-resolution mapping of H3K14-la enrichment peaks across the entire genome. It found significant upregulation of H3K14-la enrichment in the Wnt1 promoter region, subsequently revealing the molecular mechanism by which lactylation inhibits the Wnt1/β-catenin signaling pathway and impedes corneal nerve regeneration (Chen W. et al., 2026).

Although significant progress has been made with existing technologies, current research is still constrained by the limitations of detection methods. Developing more sensitive and specific antibodies is an urgent priority, as this will help reduce background noise and improve detection accuracy and reproducibility, especially in cases of low modification levels or limited sample quantities. Furthermore, applying novel epigenomic technologies like CUT&Tag to the analysis of clinical micro-samples holds great potential (Chen W. et al., 2026). Future research could attempt to utilize such technologies to analyze histone lactylation profiles in rare cells or exosomes from the cerebrospinal fluid of patients with neurological diseases, thereby mapping high-resolution lactylation modification landscapes under conditions close to clinical reality. This capability for precise detection in clinical samples is crucial for exploring the feasibility of histone lactylation as a diagnostic biomarker for neurological diseases or as an indicator for monitoring treatment response (Zhao L. et al., 2025). In summary, combining highly sensitive mass spectrometry technology with highly specific antibody tools, and promoting the application of novel detection methods in clinical samples, will greatly advance the translation of histone lactylation research in neurological diseases from basic science to clinical applications.

### 9.2 Pharmacological and genetic tools for intervening in this axis

**FIGURE 6:**
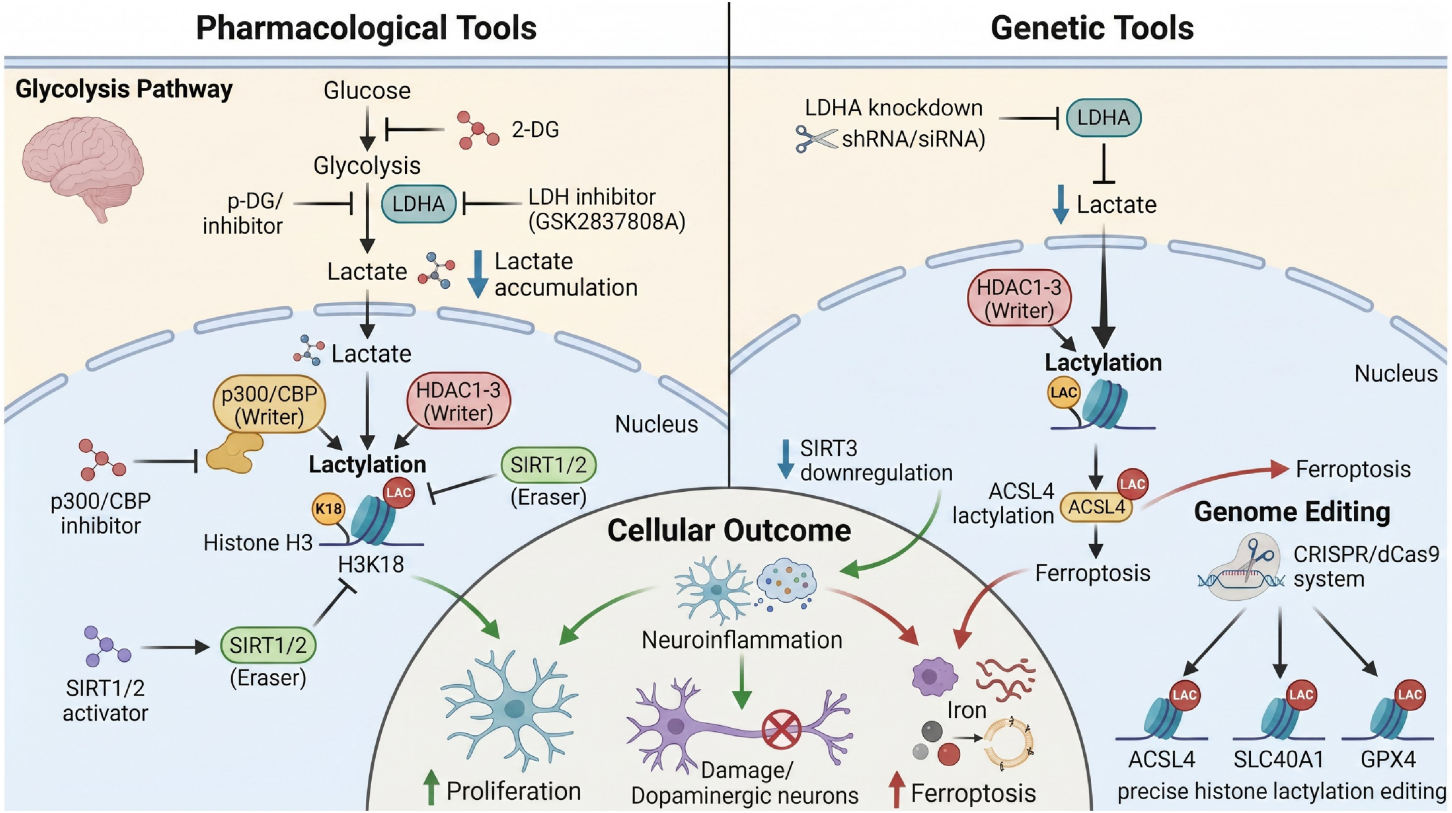
Pharmacologically, glycolysis inhibitors such as 2-DG reduce lactate accumulation in the brain, alleviating microglial proliferation, neuroinflammation, and dopaminergic neuron damage. LDH inhibitors like GSK2837808A block lactate production. The p300/CBP inhibitor suppresses H3K18-la, while SIRT1/2 activators reverse this modification. Genetically, LDHA knockdown lowers lactate and mitigates ferroptosis. HDAC1-3 enhances lactylation signaling, whereas downregulated SIRT3 increases ACSL4 lactylation to promote ferroptosis. CRISPR/dCas9 technology enables precise editing of histone lactylation at ACSL4, SLC40A1, and GPX4 loci.

In neurological diseases, targeting the glycolysis-ferroptosis axis mediated by histone lactylation provides an important direction for developing novel intervention strategies (Figure 6). Pharmacological tools are key means for validating the function of this axis. Glycolysis inhibitors, such as 2-deoxy-D-glucose, inhibit glycolysis, which can reduce lactate accumulation in the brains of PD mice, thereby alleviating microglial proliferation, neuroinflammation, and dopaminergic neuronal damage (Moreno-Yruela et al., 2022b). In an intervertebral disc degeneration model, using 2-DG to inhibit glycolysis can reduce lactate production and lactylation, subsequently mitigating ferroptosis and dysfunction in nucleus pulposus cells (Sun K. et al., 2025). In a hypoxia-induced cardiomyocyte model, 2-DG treatment reduced overall lactylation levels, the lactylation and stability of β-catenin, and alleviated ferroptosis (Zhu et al., 2026). LDH inhibitors, such as GSK2837808A, can also indirectly regulate lactylation modifications by blocking lactate production. Additionally, inhibitors targeting lactylation “writer” enzymes, such as p300/CBP inhibitors, can directly intervene in the formation of histone lactylation. For example, in rheumatoid arthritis synovial fibroblasts, silencing p300 can inhibit lactate-induced H3K18-la, thereby reducing cell proliferation (Xu et al., 2026). On the other hand, activating lactylation “eraser” enzymes, such as SIRT1/2 activators, can promote the removal of lactylation and reverse its mediated pathological effects. The application of these pharmacological tools in cellular and animal models provides evidence for elucidating the causal role of the glycolysis-lactylation-ferroptosis axis in neurological diseases.

Genetic tools offer more precise means to establish a causal relationship between histone lactylation and ferroptosis. By overexpressing or knocking down key enzymes, the levels of lactylation modifications can be specifically regulated. Overexpression of LDHA increases lactate production and lactylation, while knocking down LDHA produces the opposite effect. In intervertebral disc degeneration research, using adeno-associated virus 9-delivered siRNA to knock down LDHA effectively inhibited lactate production and lactylation, thereby improving ferroptosis and cellular dysfunction (Sun K. et al., 2025). Similarly, regulating the expression of “writer” and “eraser” enzymes such as HDAC1-3 or Sirtuin family proteins (SIRT1/2) can enhance or weaken lactylation signaling, respectively. For example, decreased expression of SIRT3 leads to increased lactylation of the ACSL4 protein, thereby promoting ferroptosis (Sun K. et al., 2025). More advanced gene editing technologies, such as the CRISPR/dCas9 system, allow for site-specific editing of histone lactylation, establishing a direct causal relationship between lactylation modifications and target gene transcription at specific genomic loci. These genetic tools enable researchers to precisely dissect the specific role of lactylation modifications in regulating the expression of ferroptosis-related genes (such as ACSL4, SLC40A1, GPX4, etc.).

## 10 Clinical translation prospects and challenges

### 10.1 Potential as diagnostic and prognostic biomarkers

Histone lactylation modification and its regulated gene expression profiles show potential as diagnostic and prognostic biomarkers in various neurological diseases. Studies indicate that specific histone lactylation modification sites, such as H3K18-la, have expression levels closely correlated with disease severity and prognosis. In AD, bioinformatic analysis based on histone lactylation-related genes successfully identified key genes like ARID5B, SESN1, and XPA. These genes are upregulated in the brain tissues of AD model mice, demonstrating potential as diagnostic biomarkers (Guo et al., 2024). Similarly, in studies of SCI, eight histone lactylation modification-related genes, including HDAC2, GCN5, and LDHA, were identified as important biomarkers. Their expression levels showed significant differences in SCI samples, and their diagnostic value was confirmed through receiver operating characteristic curve analysis (Sun Y. et al., 2025). These findings suggest that detecting specific histone lactylation modification levels or related gene expression profiles in cerebrospinal fluid or peripheral blood exosomes may provide minimally invasive or non-invasive assessment methods for reflecting neurological disease activity, neuroinflammatory status, and prognosis.

In addition to histone lactylation modification itself, its downstream-regulated gene networks also constitute an important source of biomarkers. In glioblastoma, integrated single-cell and spatial transcriptomic analyses revealed that lactylation-related gene signatures define tumor cell subpopulations with metabolic reprogramming and immunosuppressive characteristics. A nine-gene lactylation risk model constructed based on this effectively predicts patient survival prognosis (Han et al., 2025). In the anterior cingulate cortex of schizophrenia patients, ferroptosis-related genes TIMP1 and LGALS3 were identified as hub genes, and the glycolysis pathway was significantly altered (Dai et al., 2024). These studies collectively indicate that specific gene expression profiles driven by lactylation modification can reflect disease-specific molecular pathological changes and have potential as diagnostic and prognostic stratification tools.

Combining liquid biopsy with neuroimaging techniques holds promise for *in vivo* assessment of the activity of the “glycolysis-lactylation-ferroptosis” axis, thereby enabling disease subtyping and treatment response monitoring. For example, magnetic resonance spectroscopy can be used for non-invasive detection of brain lactate levels, while changes in histone lactylation modification or lactylation-regulated gene expression in peripheral blood can serve as complementary liquid biopsy indicators. In studies of sepsis and septic shock, H3K18-la levels in peripheral blood mononuclear cells have been confirmed to be significantly correlated with disease severity (e.g., Sequential Organ Failure Assessment (SOFA) score), intensive care unit length of stay, and inflammatory cytokine levels, and can serve as biomarkers for diagnosis and severity assessment (Chu et al., 2022; Di et al., 2025). In pancreatic cancer, tissue H3K18-la levels are positively correlated with serum lactate, CA19-9, and carcinoembryonic antigen levels, with an area under the curve for diagnosis reaching 0.848, demonstrating its value as a diagnostic biomarker (Hou et al., 2025). These studies lay the foundation for the future development of a precision assessment system based on combined “imaging-liquid” detection. Through multimodal information integration, dynamic monitoring of the activity of the metabolic-epigenetic axis in neurological diseases is anticipated, thereby guiding individualized treatment and predicting treatment response.

### 10.2 Opportunities and challenges as therapeutic targets

The histone lactylation-mediated glycolysis-ferroptosis axis provides rich intervention nodes for the treatment of neurological diseases. This axis encompasses multiple steps from upstream metabolic reprogramming and epigenetic modification to downstream cell death pathways (Xu et al., 2024). Targeting upstream regulation of lactate production or lactylation-modifying enzyme activity may be more fundamental than directly targeting downstream ferroptosis effectors (Gu et al., 2026a). For instance, in ischemic stroke, lactate produced by glycolysis drives immunometabolic dysfunction and neuroinflammation by inducing microglial H4K5-la, suggesting that inhibiting lactate production or lactylation modification could be an effective intervention strategy (Li Y. et al., 2026). Furthermore, targeting lactylation modifications on non-histone proteins, such as the lactylation of the key ferroptosis protein GPX4, also offers new therapeutic targets (Wang et al., 2025c). Therefore, by targeting LDHA, lactyltransferases, or delactylases, lactylation levels can be finely regulated, thereby influencing downstream ferroptosis pathways and disease progression (Chen M. et al., 2026).

However, translating this axis into effective clinical therapies faces multiple challenges. First, it is crucial to precisely distinguish between physiological and pathological lactylation modifications. Lactate, as an important energy substrate and signaling molecule in the brain, mediates lactylation modifications involved in normal brain functions such as memory formation and neuronal excitability regulation (Gu et al., 2026a). Indiscriminate inhibition of lactylation may interfere with these physiological processes. Second, blood-brain barrier permeability is a major obstacle in drug development. Most small-molecule inhibitors targeting metabolic enzymes or epigenetic modifying enzymes have difficulty effectively entering the CNS, limiting their application (Sun H. et al., 2025). Future drug design needs to develop intelligent delivery systems capable of efficiently crossing the blood-brain barrier, such as nanotechnology-based carriers. Finally, the high heterogeneity of neurological diseases demands therapeutic strategies with high cell-type specificity and spatiotemporal controllability (Alatawi et al., 2025). Developing conditionally activated prodrugs or utilizing cell-type-specific promoters to drive therapeutic gene expression are important directions for achieving precise intervention (Zhao et al., 2026). In summary, future research needs to comprehensively consider these challenges based on a deep understanding of lactylation’s role in specific neural cells and disease stages to promote the development of intelligent and precise therapeutic strategies.

## 11 Conclusion

The histone lactylation-mediated glycolysis-ferroptosis axis represents a new paradigm wherein metabolites directly regulate cell death via epigenetic mechanisms. Its discovery bridges a critical knowledge gap between metabolic reprogramming and epigenetic regulation in cell death. Moreover, it provides an integrative framework that links key disease features—including abnormal energy metabolism, oxidative stress, inflammatory responses, and cell death—into a coherent pathological network. In major neurological diseases such as AD, PD, and cerebral stroke, the active presence of this axis drives glial cell dysfunction and neuronal vulnerability, suggesting that it is not merely an accompanying phenomenon of disease progression but maybe one of the core driving forces.

Current research has preliminarily validated the neuroprotective potential of targeting key nodes of this axis (such as LDHA, p300, SIRT1), laying a solid preclinical foundation for the development of novel therapeutic strategies. The focus of future research lies in refinement and translation. First, it is essential to systematically elucidate the dynamic functional map of this regulatory axis in different brain cell types (neurons, astrocytes, microglia, oligodendrocytes) and at different stages of disease. This analysis of cell specificity and spatiotemporal specificity is a prerequisite for avoiding “one-size-fits-all” interventions and achieving precision therapy. Second, developing highly specific, blood-brain barrier-penetrating small molecule inhibitors or agonists, as well as identifying peripheral or imaging biomarkers that can reflect the activity of this axis, are essential steps for translating mechanisms into clinical applications. It is imperative to confront and overcome the significant challenges of CNS drug delivery. Finally, Balancing perspectives from different studies, it is important to recognize that this field is still in a rapid development phase. Some studies may emphasize the dominant role of lactylation in specific models, while others may suggest its crosstalk with other epigenetic modifications (acetylation, methylation) or death pathways (apoptosis, pyroptosis). Therefore, the future direction should not be to view this axis in isolation but to place it within a broader metabolic-epigenetic-cell death interaction network to explore its weight, upstream and downstream relationships, and compensatory mechanisms. In summary, in-depth exploration and targeting of the histone lactylation-glycolysis-ferroptosis axis hold promise for opening up entirely new avenues for the treatment of neurological diseases. Its transition from basic research to clinical application relies on systematic efforts to address the depth of mechanisms, the precision of tools, and the challenges of translation.
